# Multi-omics analysis detail a submicroscopic inv(15)(q14q15) generating fusion transcripts and *MEIS2* and *NUSAP1* haploinsufficiency

**DOI:** 10.1038/s41598-024-81507-7

**Published:** 2024-12-05

**Authors:** Marlene Ek, Malin Kvarnung, Maria Pettersson, Maria Johansson Soller, Britt-Marie Anderlid, Håkan Thonberg, Jesper Eisfeldt, Anna Lindstrand

**Affiliations:** 1 Department of Molecular Medicine and Surgery, Karolinska Institutet, 171 76 Stockholm, Sweden; 2 Department of Clinical Genetics and Genomics, Karolinska University Hospital, 171 76 Stockholm, Sweden; 3 Science for Life Laboratory, Karolinska Institutet Science Park, 171 65 Solna, Sweden

**Keywords:** Inversion, Short-read whole genome sequencing, Adaptive long-read sequencing, Transcriptome sequencing, *MEIS2*, Gene disruption, Gene fusion, Medical research, Molecular medicine, Neurology, Neurological disorders

## Abstract

Inversions are balanced structural variants that often remain undetected in genetic diagnostics. We present a female proband with a *de novo* Chromosome 15 paracentric inversion, disrupting *MEIS2* and *NUSAP1*. The inversion was detected by short-read genome sequencing and confirmed with adaptive long-read sequencing. The breakpoint junction analysis revealed a 96 bp (bp) deletion and an 18 bp insertion in the two junctions, suggesting that the rearrangement arose through a replicative error. Transcriptome sequencing of cultured fibroblasts revealed normal *MEIS2* levels and 0.61-fold decreased expression of *NUSAP1*. Furthermore, three fusion transcripts were detected and confirmed by Sanger sequencing. Heterozygous loss of *MEIS2 *(MIM# 600987) is associated with a cleft palate, heart malformations, and intellectual impairment, which overlap with the clinical symptoms observed in the proband. The observed fusion transcripts are likely non-functional, and *MEIS2* haploinsufficiency is the likely disease causative mechanism. Altogether, this study’s findings illustrate the importance of including inversions in rare disease diagnostic testing and highlight the value of long read sequencing for the validation and characterization of such variants.

**Supplementary Information:**

The online version contains supplementary material available at 10.1038/s41598-024-81507-7.

## Introduction

Pathogenic variants in *MEIS2* are associated with a syndrome known as “cleft palate, cardiac defects, and impaired intellectual development” (CPCMR, MIM# 600987). *MEIS2*, a homeodomain-containing protein, is part of the three amino acid loop extension (TALE) family, and functions as a transcription regulator. *Meis2* has been implicated in the development of limbs and brain in chicks, lens and retina formation in mice and *Mekada* fish, and is essential for both cardiac and neural crest development in mice^1–5^. The gene is highly intolerant to loss-of-function mutations, with a pLI score greater than 0.9, and causes disease primarily through haploinsufficiency. Most of the described *MEIS2* variants are *de novo*, although cases of inherited variants have been described. Carrier parents tend to exhibit a similar phenotype, with mosaic carriers showing milder expression^6–10^.

**Fig. 1:**
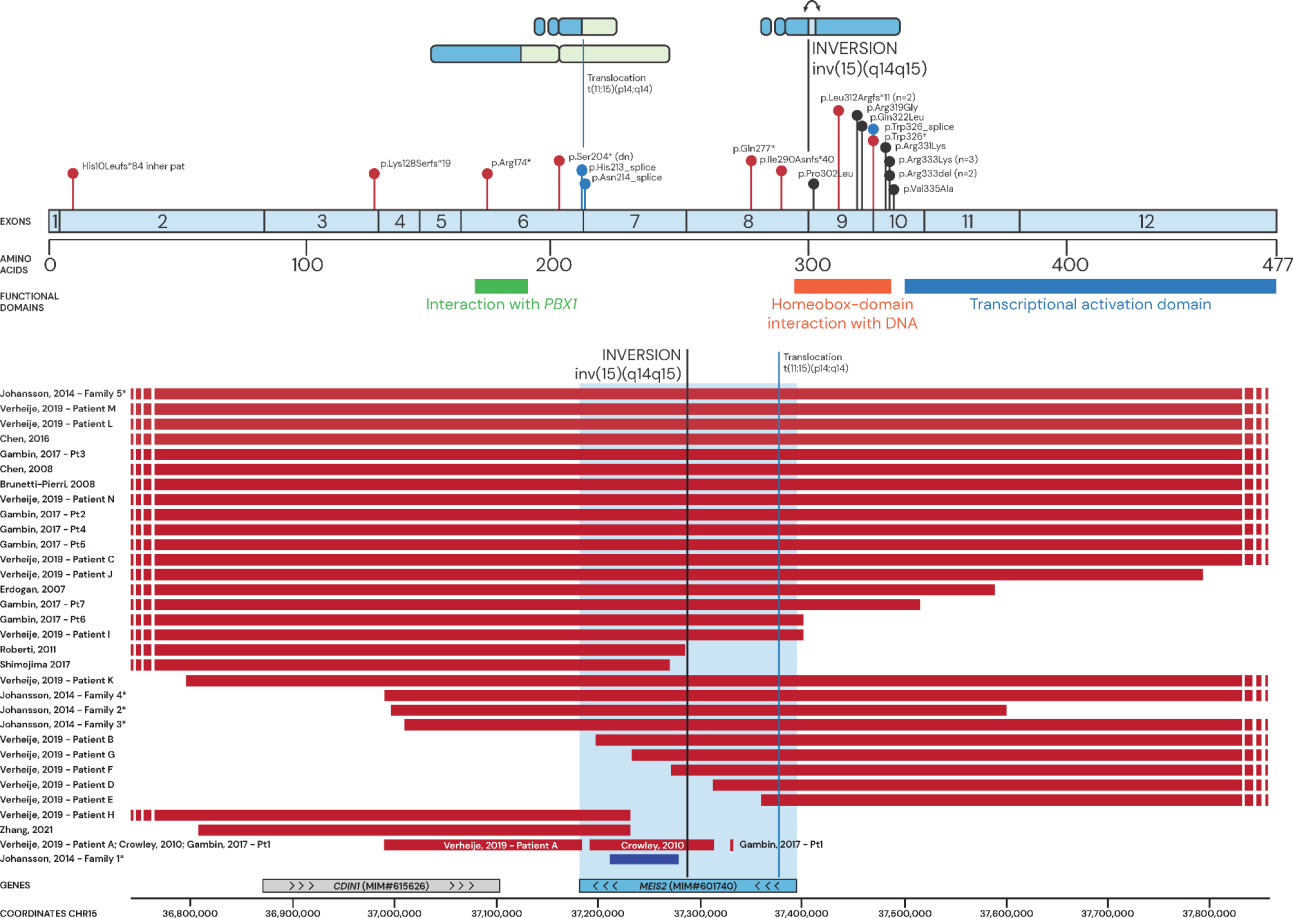
Genetic alterations in *MEIS2* in previously reported cases and our study proband. (Top) Sequence variants identified across the *MEIS2* gene. Nonsense (red), missense (black) and splice variants (blue), as well as previously reported translocation breakpoint (blue line) and our proband’s inversion breakpoint in black. (Bottom) Copy number variants (CNVs) reported in *MEIS2*, showing deletions (red), duplications (blue) translocation’s breakpoint (blue line) and our proband’s inversion breakpoint (black line). *Approximated breakpoints.

The previously reported disease-causing variants in *MEIS2* include both structural variants and sequence variants, such as single nucleotide variants (SNVs) and insertions/deletions (INDELs). The structural variants are predominantly copy number variants (CNVs), with deletions, ranging in size from 123 kb to 6.97 Mb, and one family reported with a 58 kb tandem duplication. One of the deletions was part of a *de novo* complex chromosomal rearrangement^6–8,11–18^. Notably, one case involving a balanced structural variant has been reported: a balanced reciprocal translocation between chromosomes 11 and 15, t(11;15)(p14;q14), where the breakpoint on chromosome 15 disrupts *MEIS2* in intron 6^19^. The reported SNV/INDELs include truncating, splice site, missense variants or in-frame deletions mainly within the homeodomain (Fig. 1)^9,10,18,20–27^.

We detail the genetic aberrations in a proband with cleft palate and high-functioning autism. Structural variant analysis of short-read genome sequencing revealed a 4.37 Mb inversion on chromosome 15 (Fig. 1), with breakpoints in intron 8 of *MEIS2* and intron 7 of *NUSAP1*, generating three fusion transcripts.

## Results

The proband is the first child of healthy, non-consanguineous parents. She was born after an uneventful pregnancy at 39 weeks + 6 days (birth weight 3670 g, birth length 53 cm and head circumference 35 cm). A posterior cleft palate was diagnosed early, and she experienced feeding difficulties during the first months of life. The cleft palate was surgically corrected twice, at 5 and 12 months of age. Her motor development was delayed; she sat unsupported at 9 months and walked independently at 19 months of age. She exhibited hypotonia and hypermobile joints, and her postnatal growth was slightly restricted, with a head circumference of − 2 SD and a length of − 0.5 SD at 3 years of age. Adhesions between labia majora initially raised suspicion of urethral duplication, but an ultrasound of the urinary tracts was normal. A cardiac ultrasound revealed a small atrial septal defect, which was clinically insignificant. A neuropsychological evaluation at the age of 5.5 years diagnosed her with high-function autism and her intellectual level was within the normal range. Some facial features were present, including penciled eyebrows, rounded eyes, large and protruding ears, and discrete fetal finger pads. She has two younger, healthy siblings. Genetic tests included array comparative genomic hybridization (aCGH) at 18 months of age, short-read genome sequencing analysis with in-silico gene panels for neuromuscular disorders (499 genes) and intellectual disability (885 genes) at age 7, all with normal results.

**Fig. 2:**
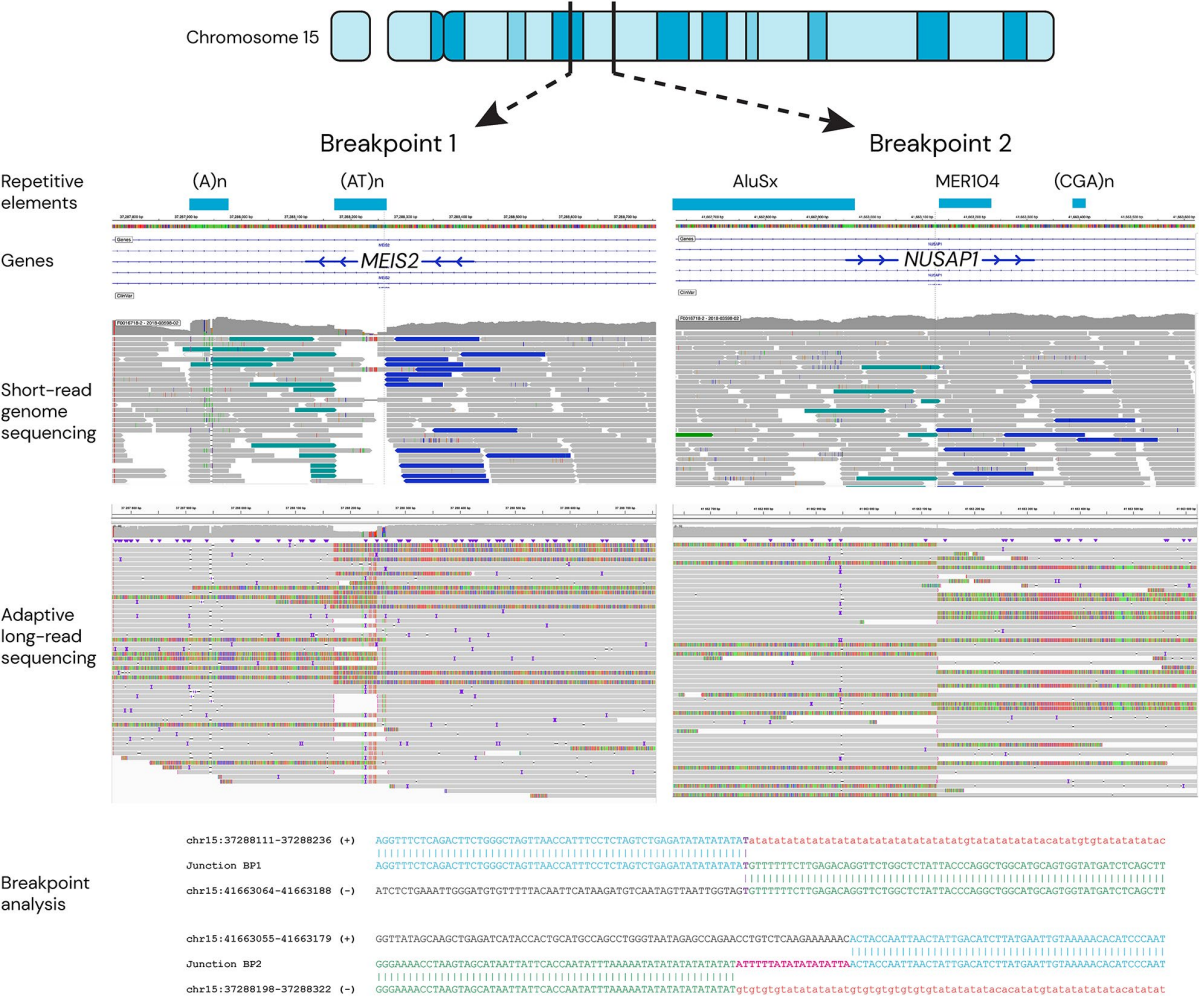
Visualization of paracentric inversion of Chromosome 15 including breakpoint analysis. Breakpoint 1 (BP1) at cytoband 15q14 disrupts MEIS2 and breakpoint 2 (BP2) at cytoband 15q15 disrupts *NUSAP1*. Repetitive elements are shown in blue boxes. Short-read genome sequencing spanning the breakpoints displays mate-pairs in teal and dark blue, respectively. Reads with rainbow pattern from adaptive long-read sequencing data indicate the two breakpoints. The breakpoint analysis with genomic positioning in GRCh19. Deleted nucleotides are shown in lower case red letters, microhomology in bold purple letters, and the inserted nucleotides in bold magenta letters.

Genome-wide structural variant analysis of the short-read genome sequencing data identified a 4.37 Mb inversion on chromosome 15. The first breakpoint (BP1), was located in intron 8 of the MANE transcript NM_170675.5 of *MEIS2*, and the second breakpoint (BP2) was found in intron 7 of *NUSAP1 *(NM_016359.5), resulting in a disruption of the homeodomain of *MEIS2*. Follow-up analysis of the parents confirmed that the variant had arisen *de novo*. The inversion was verified with adaptive long-read sequencing (Oxford Nanopore Technologies, ONT), with 34 reads supporting the breakpoints. Breakpoint junction analysis revealed a one nucleotide microhomology and a 96 bp deletion (GRCh37, chr15:g.37,288,170 − 37,288,265) in BP1. In BP2 (GRCh37, chr15:g.41,663,130), located within a MER104, there was an 18 bp insertion, of which 11 bp were templated from the adjacent sequence of the inverted segment (Fig. 2).

**Fig. 3:**
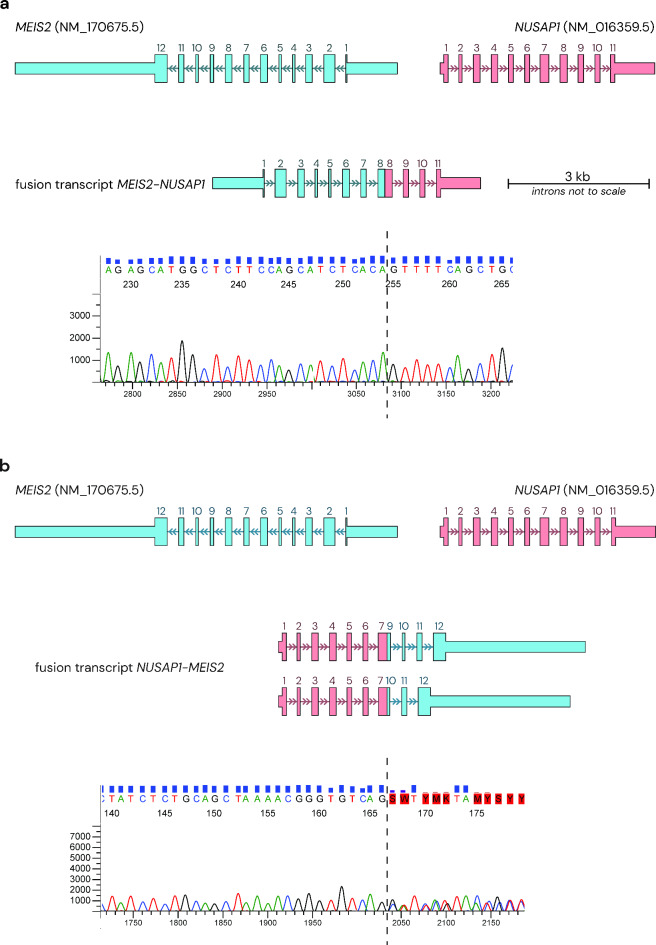
Depiction of fusion transcripts identified in our proband. *MEIS2* transcript (NM_170675.5) depicted in blue, *NUSAP1* transcript (NM_016359.5) in red. (**a**) Fusion transcript of *MEIS2*-*NUSAP1* with Sanger sequencing across the fusion junction (dashed line) of exon 8 of *MEIS2* and exon 8 of *NUSAP1*. (**b**) Fusion transcripts of *NUSAP1*-*MEIS2* with Sanger sequencing across the fusion junction (dashed line) of exon 7 of *NUSAP1* and exon 9 of *MEIS2*, as well as exon 7 of *NUSAP1* and exon 10 of *MEIS2*.

Transcriptome analysis revealed reduced expression of *NUSAP1 *(fold change 0.61) and no significant change in overall *MEIS2* expression. Additionally, three fusion transcripts were found. One between the 3’ end of *MEIS2* exon 8 and the 5’ end of *NUSAP1* exon 8, and the others between the 3’ end of *NUSAP1* exon 8 and the 5’ ends *MEIS2* exons 9 and 10 respectively. These were confirmed by Sanger sequencing of cDNA (Fig. 3).

## Discussion

We report a balanced inversion of chromosome 15, inv^15^(q14q15), detected by short-read genome sequencing and confirmed using adaptive long-read sequencing, in a female proband with cleft palate and high functioning autism. The inversion disrupted two genes, *MEIS2* and *NUSAP1*, and resulted in at least three fusion transcripts.

Long reads spanning the breakpoints allowed a detailed characterization of the rearrangement, which was not possible with short-read genome sequencing due to repetitive sequences (Fig. 2). The inversion breakpoints show evidence of a replicative error, specifically fork stalling and template switching (FoSTeS), characterized by base deletions and templated insertions^28^.

*MEIS2* is linked to cleft palate, cardiac defects, and impaired intellectual development (CPCMR, MIM# 600987), whereas *NUSAP1* has been described as tolerant of loss-of-function. The phenotypic expression varies among individuals with genetic variants in *MEIS2*, ranging from cleft palate, heart malformations, severe intellectual disability and hypotonia to cleft palate and learning problems. The more severe phenotypes appear to be associated with either large CNVs involving multiple genes, or missense variants within the homeodomain. Conversely, not all individuals with large CNVs and missense variants present with a severe phenotype, suggesting a more complex background contributing to disease expression (Supplementary Table S1). In the proband presented here, there is a clear clinical overlap with CPCMR, including cleft palate, hypotonia, delayed motor development, autism and facial features consistent with previously reported cases. The presence of a likely non-functional *MEIS2-NUSAP1* fusion transcript, along with the normal functional transcript, could be misinterpreted as two normal transcripts, potentially masking the effects of a functional haploinsufficiency. The milder phenotype in our proband, compared to previously reported *MEIS2* cases, may suggest some remaining function in the fusion gene product.

However, a recent publication reported an *NUSAP1* nonsense variant in two individuals with microcephaly, severe developmental delay, brain abnormalities, and seizures. A truncated transcript was detected that was hypothesized to evade nonsense mediated decay and have a toxic effect^29^. The phenotype in our proband did not overlap the phenotype described, but we cannot rule out a contribution from *NUSAP1*. The identified *NUSAP1-MEIS2* fusion transcripts could have a similar toxic effect as the truncated *NUSAP1* transcript described above^29^.

For the proband presented here, the diagnostic odyssey spanned over nine years, starting with aCGH and sequencing of *KMT2D* at 18 months of age. At the age of 8 years, our proband was analyzed with short-read genome sequencing without any pathogenic findings. Four years later, the genome analysis was reevaluated with a new pipeline that included structural variant detection, identifying the inversion on Chromosome 15. Currently, with genome sequencing now offered as a first-tier test to children with neurological diseases, this timeline would have been significantly shortened.

In conclusion, by applying short-read genome sequencing, adaptive long-read and transcriptome analysis a cryptic Chromosome 15 inversion was investigated. The disease-causing rearrangement, undetected by all standard clinical diagnostic tests, showcases how balanced structural variants need to be included in genetic diagnostics of syndromes and neurodevelopmental disorders. Further studies are needed to determine the clinical impact of fusion transcripts in rare diseases.

## Materials and methods

### Ethics declaration

The study was conducted in accordance with the Declaration of Helsinki and approved by the Regional Ethical Review Board in Stockholm, Sweden (protocol number 2019-04746).

Written informed consent was provided by the participant and her parents to publish this paper.

### DNA and RNA extraction

Genomic DNA was extracted from whole blood using the QIAsymphony instrument (QIAGEN, Hilden, Germany) with the QIAsymphony DSP DNA Midi Kit (cat. no. 937255, QIAGEN, Hilden, Germany), following the manufacturer’s standard protocol.

Fibroblasts were cultured from a skin biopsy in a medium composed of RPMI 1640 (1x) and Ham’s F-10 Nutrient Mixture at a 1:1 ratio, supplemented with 10% fetal bovine serum, 1% L-glutamine, and 0.2% Penicillin-Streptomycin solution (5000 U/mL). The cells were harvested by mechanical scraping from the culture flask and transferred to a PAXgene tube, and RNA was extracted using the PAXgene Blood RNA Kit (Preanalytix, cat. no. 762174, QIAGEN, Hilden, Germany) on a QIAcube Connect MDx system, following the standard protocol.

The RNA was reverse transcribed to cDNA using SuperScript VILO Master Mix (QIAGEN, Hilden, Germany). The RNA template and nuclease-free water were added to the master mix and incubated in a thermal cycler at 25 °C for 10 min, 42 °C for 60 min, and 85 °C for 5 min, followed by a hold at 4 °C. Nuclease-free water was then added, and the cDNA was stored at − 20 °C.

### Genetic analysis

Our clinical short-read genome sequencing workflow has been previously described^30–32^. To verify the inversion call, we performed adaptive long-read sequencing using the PromethION platform (ONT). The target region, spanning 32.5 Mb ([GRCh38] chr15:22,874,354 − 55,370,932) encompassed the entire inversion along with a 14 Mb buffer zone upstream and downstream of the region. Libraries were prepared from 2.76 µg of genomic DNA, following the ONT protocol ‘Ligation Sequencing gDNA (SQK-LSK114), with an average sample fragment size of 50,031 bp. The fragment size was estimated using Femto Pulse, following the protocol for the ‘Genomic DNA 165 kb Kit.’ A single PromethION R10.4 (FLO-PRO114M) flow cell was used for sequencing. The base calling was performed using the Dorado basecaller (https://github.com/nanoporetech/dorado), which was run in High Accuracy Mode (HAC). The resulting data was processed using PoorPipe (https://github.com/J35P312/poorpipe), which performs alignment using Minimap2^33^, and SV calling using Sniffles 1^34^. The inversion was manually inspected in the Integrative Genomics Viewer (IGV) using the hg19/GRCh37 reference genome. Repetitive elements were analyzed using RepeatMasker in the UCSC Genome Browser.

The effects on RNA were evaluated by whole-transcriptome sequencing of RNA isolated from cultured fibroblasts. Briefly, RNA was quantified and processed using a stranded, poly(A)-tailed kit (Illumina) before being subjected to 150 bp paired-end sequencing with approximately 150 million reads generated per sample on the Nova Seq X platform. The data was processed using the genomic medicine Sweden transcriptome pipeline Tomte (https://github.com/genomic-medicine-sweden/tomte). Briefly, the data was aligned to GRCh37 using STAR^35^, next aberrant expression events were detected by Detection of RNA Outlier Pipeline (DROP)^36^ using the default, recommended settings for OUTRIDER^37^, and fusion transcripts were detected using STAR-Fusion^38^.

### PCR and Sanger sequencing of fusion transcripts

Primers targeting the inversion breakpoints in *MEIS2*(exons 7 and 11) and *NUSAP1*(exons 7 and 8) were designed (Supplementary Table S2). Breakpoint PCR was performed using AmpliTaq Gold (Fisher Scientific, Waltham, MA, USA) with a master mix containing PCR buffer II (1x), MgCl2 (2 mM), dNTPs (100 µM), and AmpliTaq Gold (1 U). The PCR conditions included 10 min at 96 °C, followed by 35 cycles of 96 °C (30 s), 62 °C (30 s), and 72 °C (2 min), with a final extension at 72 °C for 10 min.

Amplified products were detected using the FlashGel system (Lonza) with a 100 bp–3 kb DNA marker (cat. no. 57034, Lonza) and imaged on the GenoPlex system (VWR). Sanger sequencing was performed on the normal alleles of *MEIS2* and *NUSAP1*, and the fusion transcripts *MEIS2-NUSAP1* and *NUSAP1-MEIS2* (Supplementary Table S2).

The PCR products were purified using Illustra ExoProStar, and the sequencing reaction was conducted with BigDye Terminator v3.1 (Applied Biosystems). Sequencing was done on an ABI 3500xL Genetic Analyzer (Applied Biosystems, Waltham, MA, USA).

## Electronic supplementary material

Below is the link to the electronic supplementary material.

Supplementary Material 1

Supplementary Material 2

## Data Availability

The BAM files from the adaptive long-read sequencing are available at the European Genome-phenome Archive (EGA) web portal (https://ega-archive.org/datasets/EGAS50000000632) under project ID EGAD50000000632.
