# Supplementary material for: Multi-omics analysis detail a submicroscopic inv(15)(q14q15) generating fusion transcripts and MEIS2 and NUSAP1 haploinsufficiency
Source: Sci Rep. 2024 Dec 5;14:30343. doi: 10.1038/s41598-024-81507-7 (PMC11621304; doi:10.1038/s41598-024-81507-7)
Supplement: Supplementary file 2 — Supplementary Material 2 [file 41598_2024_81507_MOESM2_ESM.docx]

**Multi-omics analysis detail a submicroscopic inv(15)(q14q15) generating fusion transcripts and MEIS2 and NUSAP1 haploinsufficiency**

Marlene Ek^1,2^*****, Malin Kvarnung^1,2^, Maria Pettersson^1,2^, Maria Johansson Soller^1,2^, Britt-Marie Anderlid^1,2^, Håkan Thonberg^1,2^, Jesper Eisfeldt^1,2,3^, Anna Lindstrand^1,2^

**Supplementary Table S2.** Primers utilized for PCR and Sanger sequencing of cDNA. Primer combinations are indicated for transcripts from the wild-type allele (blue ♦), and fusion transcripts (red ♦).

| Target | Primer sequence  (excluding M13 sequence) | Orientation | MEIS2 | NUSAP1 | MEIS2-NUSAP1 | NUSAP1-MEIS2 |
| --- | --- | --- | --- | --- | --- | --- |
| *MEIS2*  exon 7 | AACCCTTCTTCTTGGCGAGA | Forward | ♦ |  | ♦ |  |
| *MEIS2*  exon 11 | ATATGCTGCTCCTTGGCTCA | Reverse | ♦ |  |  | ♦ |
| *NUSAP1* exon 7 | ATAAGGGAGGGGTCAGGACT | Forward |  | ♦ |  | ♦ |
| *NUSAP1* exon 8 | TTTCTGGCTGGAGTCTTGGT | Reverse |  | ♦ | ♦ |  |
